# Interleukins in the pathogenesis of influenza and other acute respiratory viral infections

**DOI:** 10.1017/S0950268824001109

**Published:** 2024-09-30

**Authors:** Mykchaylo Andreychyn, Larysa Melnyk, Nataliia Zavidniiuk, Natalya Nychyk, Iaryna Iosyk

**Affiliations:** Department of Infectious Diseases with Epidemiology, Dermatology and Venerology, I. Horbachevsky Ternopil National Medical University, Ternopil, Ukraine

**Keywords:** ARVIs, influenza, interleukins, monoinfection, polyinfection, dynamics

## Abstract

Influenza and other acute respiratory viral infections (ARVIs) are among the most common human diseases. In recent decades, the discovery of cytokines and their significance in the pathogenesis of diseases has led to extensive research on these compounds in various pathologies including ARVIs. The aim of the research was to study the cytokine profile in patients with ARVIs. The cases of 30 patients were investigated. Etiological diagnosis was performed by polymerase chain reaction. Different classes of cytokines in the serum were defined by the enzyme-linked immunosorbent assay (ELISA). The level of cytokines depended on the number of pathogens. The highest levels of pro-inflammatory interleukins and the lowest levels of anti-inflammatory IL-4 were observed in patients with a combination of five or more viruses compared to those with a monoinfection. Analysis of the data showed that in the acute phase, the levels of all studied pro-inflammatory cytokines – IL-2, IL-6, and TNF-α – increased by 8, 39, and 9 times, respectively, compared to those in healthy individuals. In the acute phase of ARVI, the levels of pro-inflammatory cytokines were significantly higher and depended on the severity of the disease. The imbalance of cytokines in the serum has been established in cases of ARVIs, depending on the severity of the disease.

## Introduction

ARVIs is one of the most common infectious disease in humans; about 30%–50% of the population suffer from one of these infections at least once a year [1–3]. They are a diverse group of respiratory tract infections with similar epidemiological and clinical features, as well as development mechanisms. ARVIs are a serious issue due to the socioeconomic burden they impose on the society. The current epidemic process is represented by the circulation of different types of influenza and other ARVІs [1, 4].

In recent decades, the discovery of cytokines and their significance in the pathogenesis of diseases has led to extensive research on these compounds in various pathologies including ARVIs. The cytokine system is a general polymorphic regulatory network of mediators that controls the processes of proliferation, differentiation, apoptosis, and functional activity of the cellular elements in haematopoietic, immune, and other systems of the body [1]. The cytokines include interferons (INFs), granulocyte-colony stimulating factor (G-CSF), interleukins (ILs), chemokines, transforming growth factor, tumour growth factor group, tumour necrosis factor (TNF), and others. The main producers of cytokines are T-cells (CD4) and macrophages [5–9].

There are more than 300 identified cytokines, including IL-32. The ILs are divided into inflammatory (the most studied are IL-1α, IL-1β, IL-2, IL-6, IL-8, IL-11, and TNF-α) and anti-inflammatory (IL-4, IL-10, and IL-13) [10, 11].

Violation of the production and secretion of pro-inflammatory cytokines leads to significant defects in the immune defence and enhances the direct damaging action of microorganisms and their toxins, particularly in the lung tissue [11–13]. IL-2 is an inducible protein that begins synthesis in response to the penetration of microorganisms or tissue damage; it is essential for the development of the acute phase of inflammation [13–16]. An increase in the concentration of IL-6 is accompanied by clinical and laboratory features, such as fever, fatigue, sleep disorder, leucocytosis, and thrombocytosis; increased acute phase proteins of inflammation level; and decreased albumin level in blood. TNF-α is an active endogenous mediator involved in the development of systemic and local inflammatory and immunopathologic responses. IL-4 is secreted by antigen or T-lymphocyte mitogen and mast cells. However, IFN-γ is a key factor in determining the type of immune response [17].

Increased production or imbalance of pro-inflammatory cytokines is important in the pathogenesis of pneumonia by intensified aggregation of leukocytes to the vascular epithelium, stimulation of pro-coagulating activity, effector cells involvement in the area of inflammation, which increases the patho-immune process and leads to cytokine-mediated lung injury [18, 19]. There is a theory named the ‘cytokine explosion,’ which posits that the cytokines in severe influenza (including avian influenza) quickly fill the infected lung tissue, leading to patients’ death [20–25].

In patients with influenza and other ARVIs, an increased content of IFN-α and IL-2 in the serum in the acute stage of the disease was established. It was caused by antigenic stimulation and inflammation [11, 24].

The aim of the research was to investigate the dynamics of cytokines level in the blood of patients with ARVIs of various aetiologies.

## Methods

The cases of 30 patients, aged 18–58 years, who were treated for influenza and other ARVIs during the increase in seasonal morbidity (2022–2023) were studied. There were 18 men and 12 women. The patients were distributed according to the severity of ARVIs: severe course in 16 (53.3%) patients and moderate course in 14 (46.7%) patients. In seven patients (23.3%), ARVIs was complicated by pneumonia. Representation of patients by age is as follows: 9 (30.0%) patients under 20 years old, 13 (43.3%) patients aged 21–40 years, and 8 (26.7%) patients aged 41 years and over. Most patients (22) were observed from the first day to the fourth day after the onset of illness and 8 after the fifth day. Fifteen healthy persons served as the control group (the same age and gender).

The decryption of the etiological diagnosis of influenza and other ARVIs was performed by polymerase chain reaction (PCR) for respiratory virus group. The cytokines of different classes (IL-2, IL-4, IL-6, and TNF-α) in the serum were determined by the enzyme-linked immunosorbent assay (ELISA) using test kits produced by Vector-Best. The research was performed in dynamics; the first blood sample was taken on the day of admission and the second on the seventh to tenth days. The test results were analysed in the Interdepartmental Research Laboratory and Laboratory Studies of Infectious Diseases of TNMU.

### Statistical analysis

SPSS v.28.0 for Windows was used for statistical evaluation of the attained results. The groups of parametric variables were compared using Student’s *t*-test and analysis of variance. The groups of nonparametric variables were compared using the Mann–Whitney *U*-test. In addition, the Shapiro–Wilk test was used for parametric and nonparametric differentiation. The results were presented as mean ± SEM. A *p* value of <0.05 was considered to be statistically significant.

## Results

A complex PCR examination detected influenza and other ARVIs pathogens in all 30 patients. The spectrum of pathogens included influenza virus A/H1N1sw (in 10 patients), influenza B (in 6), parainfluenza virus-1-4 (in 26), RS-virus (in 14), coronavirus (in 15), metapneumovirus (in 12), and rhinoviruses (in 5 individuals). Various combinations of two to eight pathogens at the same time were evidenced in 22 patients.

The frequency of positive results depended on the schedule of material collection for research. The research efficiency by PCR was 82.2% up to the fifth day of illness, and only 50.0% from the sixth day onwards (*p* < 0.05).

In the acute phase of the disease, all the studied concentrations of pro-inflammatory cytokines were higher than those observed in the healthy individuals. The indicators of IL-2 ranged from 28.56 ± 3.53 pg/mL in cases of rhinovirus infection to 54.60 ± 8.83 pg/mL in influenza A (rate 4.18 ± 0.15; *p* < 0.001). The level of IL-6 was 26.14 ± 7.20 pg/mL for influenza and 32.60 ± 3.68 pg/mL for coronavirus infection (rate 0.68 ± 0.04; *p* > 0.05). The level of TNF-α in the acute period ranged from 23.93 ± 4.23 pg/mL for influenza B to 32.40 ± 5.24 pg/mL for influenza A (rate 2.95 ± 0.04; *p* > 0.05). The level of anti-inflammatory cytokine IL-4 decreased in the acute stage of the disease. The indicators ranged from 0.81 ± 0.33 pg/mL for influenza B to 1.39 ± 0.23 pg/mL for MS infection (rate 2.17 ± 0.02; *p* > 0.05).

In the dynamics of the disease from the seventh to the tenth day, the concentration of pro-inflammatory cytokines decreased, although it did not reach a high level. The range of IL-2 was 8.85 ± 0.85 pg/mL for parainfluenza and 11.79 ± 1.17 pg/mL for rhinovirus infections (*p* < 0.01); IL-6 1.95 ± 0.32 pg/mL for influenza B and 2.89 ± 0.73 pg/mL for rhinovirus infections; TNF-α 3.86 ± 0.38 pg/mL for influenza A and 4.89 ± 0.44 pg/mL for сoronavirus infection. The level of anti-inflammatory cytokine IL-4 in the same disease period increased, exceeding the normal rate by 1.20–1.93 times. The indicators ranged from 2.70 ± 0.17 pg/mL for influenza B to 4.19 ± 0.54 pg/mL for MS infection (*p* < 0.001).

**Figure 1.**
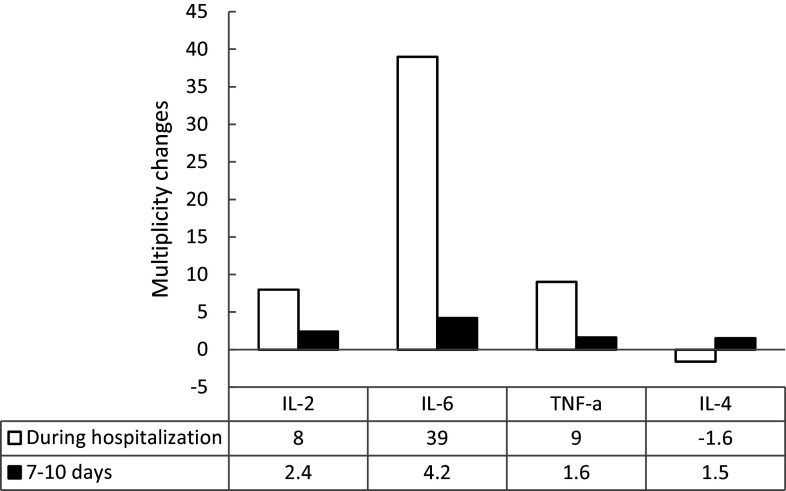
Inflammatory changes of (IL-2, IL-6, TNF-α) and anti-inflammatory (IL-4) cytokines in patients with ARVIs according to the established norm.

In the acute phase of the disease, levels of pro-inflammatory cytokines – IL-2, IL-6, and TNF-α – increased (*p* < 0.001), by about nine times for IL-2, IL-8, IL-6th 39, and TNF-α compared to those in healthy individuals. The content of anti-inflammatory IL-4 decreased by 1.6 times (*p* < 0.001) (Figure 1).

In the early convalescence period, the interleukin concentration decreased in all patients (*p* < 0.001) but was still high; it did not reach the levels observed in healthy individuals (Table 1).

**Table 1.** Concentration of IL-2, IL-6, TNF-α, and IL-4 in the serum of patients with ARVIs (*M* ± *m*)

Unit (pg/mL)	Healthy persons (*n* = 15)	Patients	
		During hospitalization (*n* = 30)	After hospitalization (*n* = 30)
IL–2	4.18 ± 0.15	34.39 ± 3.62^a^	8.51 ± 0.77^a^ ^,^^b^
IL–6	0.68 ± 0.04	26.83 ± 2.58^a^	2.95 ± 0.43^a^ ^,^^b^
TNF–α	2.95 ± 0.04	27.10 ± 2.13^a^	4.62 ± 0.31^a^ ^,^^b^
IL–4	2.17 ± 0.02	1.30 ± 0.13^a^	3.46 ± 0.26^a^ ^,^^b^

a Significant difference (*p* < 0.01–0.001) of performance compared to virtually healthy persons.

b Between parameters in hospitalization and early convalescence periods.

At the same time, the level of anti-inflammatory IL-4 increased by about 2.6 times of the original level. In the recovery period, it was even excessive by 1.5 times of the level observed in healthy individuals, up to 3.40 ± 0.25 pg/mL (*p* < 0.001), which evidenced a reduction in inflammation and a high level of activation of anti-inflammatory mechanisms of immune defence in convalescence [24].

A dependency of cytokines on the number of pathogens was also established. At the time of hospitalization, the highest levels of pro-inflammatory interleukins were observed for IL-2, IL-6, and TNF-α, whereas the lowest levels were observed for inflammatory IL-4 identified in patients with combined viruses in quantities of two or more compared to monoinfection. No significant differences were found between the rates after treatment.

Some dependency of pro-inflammatory and anti-inflammatory interleukins on the patient’s age and gender was established. The most characteristic changes of interleukin were observed in individuals aged 20 years (IL-2 and IL-4), 21–30 years (IL-4), and 41 and older (IL-2 and IL-6). TNF-α content did not depend on age. The level of IL-2 in women of all ages during the acute period of the disease was higher than that in men: 39.7 ± 4.1 to 25.1 ± 2.7 pg/mL (*p* < 0.01); IL-6: 29.2 ± 3.1 to 20.7 ± 2.9 pg/mL (*p* < 0.05). No difference in value was found between IL-4 and TNF-α.

The dependence of disease parameters on the severity of disease is presented in Table 2.

**Table 2.** The level of cytokines in patients with ARVIs depending on disease severity (*M* ± *m*)

Unit (pg/mL)	Healthy persons (*n* = 15)	Moderate form of the disease (*n* = 14)		Severe form of the disease (*n* = 16)	
		Before treatment	On the seventh to tenth days	Before treatment	On the seventh to tenth days
	1	2	3	4	5
IL–2	4.18 ± 0.15	18.45 ± 1.87	6.65 ± 0.71	48.34 ± 4.15	10.15 ± 1.18
		*p*_1–2_ < 0.001	*p*_1–3_ < 0.001	*p*_1–4_ < 0.001	*p*_1–5_ < 0.001
			*p*_2–3_ < 0.001	*p*_2–4_ < 0.001	*p*_3–5_ < 0.01
					*p*_4–5_ < 0.001
IL–6	0.68 ± 0.04	14.24 ± 0.71	3.77 ± 0.76	37.84 ± 2.52	2.23 ± 0.41
		*p*_1–2_ < 0.001	*p*_1–3_ < 0.001	*p*_1–4_ < 0.001	*p*_1–5_ > 0.05
			*p*_2–3_ < 0.001	*p*_2–4_ < 0.001	*p*_3–5_ > 0.05
					*p*_4–5_ < 0.001
TNF–α	2.95 ± 0.04	15.94 ± 0.97	5.00 ± 0.56	36.92 ± 1.44	4.29 ± 0.31
		*p*_1–2_ < 0.001	*p*_1–3_ < 0.001	*p*_1–4_ < 0.001	*p*_1–5_ < 0.05
			*p*_2–3_ < 0.001	*p*_2–4_ < 0.001	*p*_3–5_ > 0.05
					*p*_4–5_ < 0.001
IL–4	2.17 ± 0.02	1.44 ± 0.202	3.58 ± 0.34	1.18 ± 0.15	3.34 ± 0.40
		*p*_1–2_ < 0,001	*p*_1–3_ < 0,001	*p*_1–4_ < 0.001	*p*_1–5_ < 0.01
			*p*_2–3_ < 0,01	*p*_2–4_ > 0.05	*p*_3–5_ > 0.05
					*p*_4–5_ < 0.001

In the acute phase of ARVIs, the levels of pro-inflammatory cytokines increased depending on the severity of the disease and were higher in severe cases compared to moderate cases (*p* < 0.01–0.001). The indicators of these cytokines in the period of convalescence did not depend on the severity and did not make a difference except for IL-2, which returned to normal slowly. Anti-inflammatory IL-4 changes were independent at the different periods of disease severity.

The concentrations of pro-inflammatory IL-2, IL-6, and TNF-α, as well as the anti-inflammatory IL-4 in patients with ARVIs complicated by pneumonia did not differ from those in patients with an uncomplicated course of illness, which is based on the data calculated by standard deviation and nonparametric criterion.

The association of clinical symptoms of ARVIs with the concentration of interleukins was studied. The concentration of the IL did not depend on the severity of fever. Regarding the duration of fever, a medium correlation was detected for IL-2 (*r* = −0.41) and IL-6 (*r* = −0.34), weak for IL-4 (*r* = 0.26), and average for TNF-α (*r* = 0.56).

The duration of cough had an inverse association with IL-2 (*r* = −0.46) and IL-4 (*r* = −0.31) and a direct association with IL-6 (*r* = 0.39–0.63 at different severities of ARVI) and TNF-α (*r* = 0.35).

A direct association was found between the number of hospital days and the concentration of pro-inflammatory cytokines: IL-2 (*r* = 0.38), IL-6 (*r* = 0.63), TNF-α (*r* = 0.49) and an inverse weak association for anti-inflammatory interleukin IL-4 (*r* = −0.24).

The association of IL-4 with all studied parameters was reversible, and with TNF-α, it was direct. IL-2 had a reverse association with fever and cough and a direct association with day and duration of hospitalization. IL-6 had an inverse association with duration of fever and a direct association with duration of cough and time of hospital stay.

A direct correlation between the inflammatory cytokines – high for TNF-α → ІL-6, medium for TNF-α → ІL-2 and ІL-2 → ІL-6 – was established. On the seventh to tenth day, only a medium correlation of ІL-2 → ІL-6 was evidenced. Other associations were weak (*r* < 0.3) and reversible for IL-4.

## Discussion

Therefore, in patients with acute respiratory diseases in the acute phase, the production of pro-inflammatory cytokines increased and anti-inflammatory cytokines reduced. Most expressive changes were evidenced in pro-inflammatory IL-2. It meant that the high intensity of inflammatory and immune processes that accompany diseases of varying severity could be considered as an adequate body response. In the recovery period, the synthesis of IL-2, IL-6, and TNF-α decreased, along with the regression of clinical symptoms, showing a tendency towards normalization. Although in the early convalescence period, it was still high, which indicated incompleteness of inflammation. The level of anti-inflammatory IL-4 decrease in the acute period; the dynamics of the disease also had a tendency to stabilization by increasing, especially in severe illness, which may have proved that immune response was restructuring towards the anti-inflammatory and immune-inflammatory correction of body response through cytokines [8, 19, 20, 22].

Thus, the research on the physiological and pathophysiological mechanisms of cytokines is promising and allows expanding the knowledge of the pathogenesis of ARVIs. It also offers benefits for identifying diagnostic and prognostic markers of inflammation in these diseases.

## Conclusion

In the acute phase of ARVIs, an increase in pro-inflammatory cytokines in the serum was established: IL-2, IL-6, and TNF-α by 8, 39, and up to 9 times, respectively, compared to the relevant indicators in healthy subjects (*p* < 0.001). The level of these cytokines increased depending on the severity of the disease and the number of pathogens in combination. In the early convalescence, some decrease in these indicators (*p* < 0.01–0.001) was evidenced, but normal rates were not observed in all cases.

The indicators for the anti-inflammatory IL-4 significantly decreased by about 1.6 times in the acute period compared to norm (*p* < 0.01); the level of interleukin did not depend on the severity of the disease. Inhibition was more significant under the influence of a combination of viruses. In the early convalescence period, cytokine concentrations increased by about 2.6 times of the original level and exceeded the level of healthy individuals by 1.5 times.

It has been established that a direct correlation between the inflammatory cytokines TNF-α → ІL-6 is high, TNF-α → ІL-2 is medium, and ІL-2 → ІL-6 is medium. Other associations are weak (*r* < 0.3), and reversible for IL-4.

## Data Availability

The datasets generated and/or analysed in the current study are not publicly available due to them containing personal data.
